# Fluorescent Protein Expressing *Rickettsia buchneri* and *Rickettsia peacockii* for Tracking Symbiont-Tick Cell Interactions

**DOI:** 10.3390/vetsci3040034

**Published:** 2016-11-17

**Authors:** Timothy J. Kurtti, Nicole Y. Burkhardt, Chan C. Heu, Ulrike G. Munderloh

**Affiliations:** Department of Entomology, University of Minnesota, 1980 Folwell Avenue, Saint Paul, MN 55108, USA; burkh032@umn.edu (N.Y.B.); heuxx012@umn.edu (C.C.H.); munde001@umn.edu (U.G.M.)

**Keywords:** ticks, endosymbionts, *Rickettsia buchneri*, *Rickettsia peacockii*, transformation, shuttle vector, *Himar 1* transposition, green fluorescent protein, mCherry fluorescent protein, spectinomycin, rifampin

## Abstract

Rickettsiae of indeterminate pathogenicity are widely associated with ticks. The presence of these endosymbionts can confound a One Health approach to combatting tick-borne diseases. Genomic analyses of symbiotic rickettsiae have revealed that they harbor mutations in gene coding for proteins involved in rickettsial pathogenicity and motility. We have isolated and characterized two rickettsial symbionts—*Rickettsia peacockii* and *R. buchneri*—both from ticks using tick cell cultures. To better track these enigmatic rickettsiae in ticks and at the tick-mammal interface we transformed the rickettsiae to express fluorescent proteins using shuttle vectors based on rickettsial plasmids or a transposition system driving insertional mutagenesis. Fluorescent protein expressing *R. buchneri* and *R. peacockii* will enable us to elucidate their interactions with tick and mammalian cells, and track their location and movement within individual cells, vector ticks, and host animals.

## 1. Introduction

Rickettsiae of indeterminate pathogenicity are often detected in ticks, raising questions about their infectivity for vertebrate hosts. Some are labeled as non-pathogenic beneficial endosymbionts (e.g., *Rickettsia buchneri*) [1,2,3,4] while others are considered potential pathogens (e.g., “*Candidatus* R. amblyommii”) [5,6]. Rickettsiae are maintained in nature via horizontal (infectious) transmission and/or vertical (transovarial) transmission. The current model specifies that horizontal transmission of rickettsiae during the bloodmeal of an infected arthropod feeding on a vertebrate host favors the evolution of pathogenic rickettsiae while non-pathogenic rickettsiae transmitted transovarially evolve to become mutualists [7]. Major advances are being made in our understanding of virulence mechanisms and pathogenesis of horizontally transmitted rickettsiae [7,8]. In contrast, we have limited understanding of the host parasite interactions and transmission mechanisms of endosymbiotic rickettsiae that are transmitted transovarially.

Non-pathogenic rickettsial endosymbionts, such as *R. buchneri* and *Rickettsia peacockii*, are transtadially and transovarially transmitted in ticks and are mainly associated with ovarian tissues. Both endosymbionts are non-infectious for vertebrates and mammalian cells [1,3]. Surprisingly, they are closely related to pathogenic rickettsiae that are horizontally transmitted to vertebrate hosts by ticks; *R. peacockii* is closely related to *R. rickettsii* [9], and *R. buchneri* is closely related to *Rickettsia monacensis* [3,10]. Genomic analyses of these endosymbionts have identified gene mutations that dampen their virulence and ability to cause cytopathic effects [2,11]. Both endosymbionts possess genomes extensively rearranged following introduction of multiple copies of transposons, which led to numerous mutations via recombination between transposon copies and deletion or disruption of several genes important to rickettsial pathogenicity, restricting them to the tick [2,11]. Both species are maintained in nature in ticks via transovarial transmission, and the ovaries of these ticks harbor numerous rickettsiae [1,3,12]. Interstitial cells as well as developing oocytes are colonized and fecundity is not adversely affected [1,13]. Other organs in the tick are rarely infected, supporting the observation that these species are solely transmitted transovarially, but not horizontally. The study of these enigmatic rickettsiae is facilitated by their isolation and propagation in tick cell culture systems, demonstrating that they have retained the ability to invade and replicate in host cells [3,9,10,14].

Tick endosymbionts are closely related to pathogenic rickettsiae that can be horizontally transmitted to vertebrate hosts by ticks [3,10]. Pathogenic rickettsiae cause disseminating infections in both vertebrate hosts and ticks and a reduction in tick fecundity [15,16,17,18]. However, strains of pathogenic rickettsial species (e.g., *Rickettsia rickettsii* the agent of Rocky Mountain spotted fever) can vary considerably in their infectivity for vertebrates and ticks [15,16]. Avirulent *R. rickettsii*, e.g., strain Iowa, retain the ability to disseminate and persist in the tick, infect ovaries, and be transmitted transovarially [15,19]. Such avirulent strains need to be clearly delineated from nonpathogenic rickettsial endosymbionts.

A goal of our research is to study the interaction between rickettsial endosymbionts and their host cells in vivo and in vitro in order to characterize the cellular processes involved in rickettsial symbiosis with ticks. Recent advances in the genetic transformation of rickettsiae provide us with new research tools for studies on endosymbiotic and other enigmatic rickettsiae [20]. These techniques enable us to genetically manipulate these rickettsiae and track the events involved with rickettsial endosymbiosis (e.g., cellular adherence, entry, and motility). Here we describe the transformation of *R. buchneri* and *R. peacockii* to express fluorescent proteins (GFPuv or mKate), using a shuttle vector based on the plasmid pRAM18 of *Candidatus* Rickettsia amblyommii [21]. Transformed symbionts grew and brightly expressed fluorescent reporter proteins in host cells. Second, we used a transposition system based on a hyperactive Himar1 transposase for driving insertional mutagenesis of mCherry in *R. peacockii* [22]. The flanking genomic insertions were sequenced to confirm transposition.

## 2. Materials and Methods

### 2.1. Tick Cell Lines

Cell lines ISE6 and IRE11 from embryos of *I. scapularis* (ISE 6) [23] and *Ixodes ricinus* (IRE 11) [10], respectively, were maintained in L15C300 medium [24] supplemented with fetal bovine serum (FBS, 5%), tryptose phosphate broth (TPB, 5%; Difco), and lipoprotein concentrate (LPC, 0.1%; MP Biomedicals). Cultures were incubated at 32 or 34 °C.

### 2.2. Rickettsiae

*R. buchneri* (str ISO7^T^) (clone B8), isolated from ovaries of an *I. scapularis* female [3], was grown using IRE11 [10]. Cultures were maintained in ambient air and incubated at 26 or 28°C in L15C300 medium supplemented with FBS (10%), TPB (5%), and LPC (0.1%). Every four weeks, 1 mL (~2 × 10^6^ cells) of infected cells were transferred to 5 mL of uninfected IRE11 cells (~10^7^ cells) in a 25 cm^2^ flask.

*R. peacockii* (str. Rustic) [9] was maintained in ISE6 using L15C300 medium supplemented with FBS (10%), TPB (5%), LPC (0.1%), HEPES (25 mM), and NaHCO3 (0.25%). Infected cultures were incubated at 32 or 34 °C, and 0.1 mL of an infected cell suspension (~2 × 10^5^ cells) was added to a fresh cell layer (5 mL of uninfected ISE6 cells, ~10^7^ cells, in a 25 cm^2^ flask) every ~3 weeks.

### 2.3. Plasmid Constructs

The plasmid constructs that we used in this study are listed in Table 1.

### 2.4. Plasmid Transformation of R. buchneri and R. peacockii

Shuttle vector pRAM18dRGA [21] was used to transform *R. buchneri* and *R. peacockii*. The shuttle vector was developed using pRAM18 originally found in *Candidatus* Rickettsia amblyommii [21]. The vector carries a selection cassette encoding *R. prowazekii arr-*2 rifampin resistance gene (*rpsLp-arr-2_Rp_*) or a gene for spectinomycin and streptomycin resistance (*aadA*) and a reporter gene encoding a green (GFP_uv_) (Becton Dickenson, Palo Alto, CA, USA) [25] or far red (mKate) (DNA 2.0, Newark, CA, USA) [26] fluorescent protein.

*Rickettsia buchneri* was transformed with pRAM18dRGA encoding rifampin resistance and GFPuv fluorescence by electroporation using procedures described previously [21]. Briefly, rickettsiae were released from IRE11 cells using shear created by forcing cells suspended in medium through a 25 G needle attached to a 5 mL syringe. The lysate was filtered through a 1.5 µm pore size filter, washed twice (13.6 rcf × 4 min × 4 C) in 300 mM sucrose, concentrated into 50 µL of 300 mM sucrose with 1 µL (1–2 µg) of pRAM18dRGA, and transferred to a cuvette (Gene Pulser Cuvette, 0.1 cm gap electrode, Bio-Rad Laboratories, Inc., Hercules, CA, USA). Cuvettes containing rickettsia-plasmid preparations were held on ice for 15 min and then pulsed once (1.8 kV, 200 ohms, 25 µF, ~5 ms) using a Gene Pulser II electroporation apparatus. Electroporated rickettsiae were transferred to a 2 mL microfuge tube containing IRE11 cells (2 × 10^6^ cells in 1.5 mL), centrifuged at 5000 rcf for 5 min and incubated at room temperature for 30 min. Finally, the *R. buchneri*-IRE11 suspension was transferred to a vented cap flask (12.5 cm^2^) in 5 mL L15C300 supplemented as described, and incubated at 26 °C in candle jars having an atmosphere of approximately 3% CO_2_ and 17% O_2_. After three days, 0.8 µg/mL of Rifampin was added. The medium was changed weekly while maintaining continuous rifampin selection. Cultures were monitored for presence of green fluorescent rickettsiae by epifluorescence microscopy using an inverted Nikon Diaphot fitted with a Sapphire GFP filter. Cultures with transformed *R. buchneri* were noted three to four weeks later and subcultured one to twothrmonths post electroporation. After four serial transfers transformed *R. buchneri* were maintained in ambient air using medium containing 0.8 µg/mL rifampin.

*Rickettsia peacockii* was transformed using shuttle vector pRAM18dSGA encoding spectinomycin resistance (*aadA*) and the fluorescent proteins mCherry (DNA 2.0) [27] or GFP_uv_ [21,24]. Cell free *R. peacockii* were prepared using approximately 0.2 mL of sterile rock polishing grit (60/90 grit silicon carbide; Lortone, Inc., Mukilteo, WA, USA) in a 2 mL microfuge tube to which the cell suspension was added. The grit-cell suspension was vortexed for 30 s and the lysate filtered through a 2 µm syringe filter. Cell free *R. peacockii* were washed and electroporated as given above for *R. buchneri*, excepting cultures were incubated at 34 °C. Spectinomycin (10 µg/mL) was added three days later. Cultures were monitored for presence of fluorescent rickettsiae as described above, and subcultured two months post electroporation. Transformants were maintained using spectinomycin-supplemented medium as described for wild-type rickettsiae.

We maintained both transfomants of both species by mixing infected cells or cell free rickettsiae with uninfected host cells. Centrifugation (13,600 rcf for 2.5 min; or 170 rcf, 3 min; 1 mL of suspension on a “dry” cell layer) of cell free *R. buchneri* with target host cells greatly enhanced the infection rate.

### 2.5. Transposon Mutagenesis (HIMAR1 A7) of R. peacockii

We used the 8423 bp plasmid pCis mCherry-SS HIMAR1 A7 to transform *R. peacockii* [20,22]. This cis-construct included both transposase and transposon encoded on a single plasmid, in order to improve efficiency of transformation. It encodes the A7 hyperactive mutant of the HIMAR1 transposase controlled by the *Anaplasma marginale* transcriptional regulator promoter, tr, that is well-expressed in both tick and mammalian cells [28], and a transposon carrying the Am tr promoter driving expression of mCherry or GFPuv and spectinomycin resistance. This promoter works efficiently in our hands for expression of transgenes in the genera *Anaplasma*, *Ehrlichia*, and *Rickettsia*. The coding genes are positioned between left and right HIMAR transposon repeats that are recognized by the transposase to facilitate excision followed by random insertion into genomic target sites containing TA dinucleotides [29]. 1 µg of *himar1* plasmid DNA was mixed with 50 µL of host cell free *R. peacockii* and transferred to 1 mm gap electroporation cuvettes, incubated on ice for 15 min, and electroporated at 2.4 kV, 25 mF, 400 ohms, and ~8 ms. Electroporated rickettsiae were transferred to a 2 mL microfuge tube containing ISE6 cells (2 × 10^6^ cells in 1.5 mL), centrifuged at 10,000 rcf for 5 min and incubated at room temperature for 15 min. Finally, the *R. peacockii*-ISE6 pellet was resuspended in 5 mL of medium, transferred to a flask (25 cm^2^) and incubated at 34 °C. Spectinomycin (5–10 µg/mL) was added two to three days later. Cultures with transformed *R. peacockii* were noted eight weeks later and subcultured three months post electroporation as described for wild-type rickettsiae. Transformants were maintained under continuous selection using spectinomycin.

### 2.6. Cloning and Sequencing of Transposon Integration Sites

Genomic insertion sites were determined by plasmid rescue cloning as previously described [22,30]. Briefly, *R. peacockii* mCherry himar transformant genomic DNA was digested with EcoRI and HindIII (NEB, Beverly, MA, USA), purified by phenol/chloroform extraction, and ligated into dephosphorylated, EcoRI or HindIII-cut pMOD vector. The plasmid was transformed into *E. cloni* Elite electrocompetent cells (Lucigen, Middleton, WI, USA), and clones containing the transposon were selected on 50 μg/mL spectinomycin/streptomycin YT agar plates. Plasmid DNA from red-fluorescent, spectinomycin/streptomycin-resistant clones was isolated with the High Pure Plasmid Isolation kit (Roche, Madison, WI, USA) as per manufacturer’s protocol, checked by restriction digest (EcoRI or HindIII) for presence of inserts, and sequenced with primers reading out from the transposon at the University of Minnesota Genomics Center Sequencing and Analysis Facility.

### 2.7. Microscopy

Cultures were observed weekly by phase-contrast microscopy to assess culture confluency and the presence and relative abundance of rickettsiae. Cultures were periodically sampled for the presence of rickettsiae using cells deposited onto slides by means of a cytocentrifuge (Cytospin; Shandon, Pittsburgh, PA, USA), fixed in methanol and stained with Giemsa. Cell layers were additionally monitored for presence of fluorescent rickettsiae by epifluorescence microscopy using an inverted Nikon Diaphot fitted with a Sapphire GFP filter or a TRITC Filter (Rhodamine)/Dil/Cy3 (Chroma Technology, Bellows Falls, VT, USA). Suspended cells on microscope slides were examined using a Nikon Eclipse E400 fitted with FITC and TRITC filters.

### 2.8. Preparation of Genomic DNA for PFGE and Southern Blot Analysis

Cell free rickettsiae were prepared as described above, embedded in agarose (1%, InCert low melting point) and lysed in situ with proteinase K and sodium lauryl sarcosine in 0.5 M EDTA [21]. Released DNA was separated by pulsed-field gel electrophoresis (PFGE) on a Chef Mapper XA System (Bio-Rad) with 0.5× TBE using the Auto setting in CHEF mode. Chef Mapper XA System parameters were: DNA size range of 10 kbp to 100 kbp, a gradient of 6 V/cm, an angle of 120°, a linear ramping factor, a calibration factor of 1, an initial switch time of 0.47 s, and a final switch time of 8.53 s. The total run time was 20.18 h.

For Southern blotting, pulsed-field gels were depurinated and DNA transferred onto a Zeta Probe GT genomic membrane (Bio-Rad) [21]. To detect *R. buchneri* plasmids, blots were hybridized at 55 °C overnight with digoxigenin-labeled parA probes specific for pREIS1, 2, 3, and 4. To detect the native plasmid pRPR in *R. peacockii*, blots were hybridized with digoxigenin-labeled pRM6, which encodes the conserved chaperonin Hsp2 on the *Rickettsia monacensis* plasmid pRM (Baldridge et al. 2010). To detect *gfp*_uv_ encoded on the shuttle vector pRAM18dRGA (*R. buchneri*) or pRAM18dSG (*R. peacockii*) in transformants, blots were labeled with digoxigenin-labeled *gfp_uv_*, washed at 55 °C, and reacted with anti-digoxigenin Fab fragments conjugated to alkaline phosphatase (Anti-Digoxigenin-AP, Fab fragments; Roche) and detected with CDP-Star (Roche). Blots were exposed to Kodak X-OMAT AR film, or fluorescence from blots was captured with the Infinity 3 camera with Infinity Analyze Software version 5.0 (Lumenera Corporation, Ottawa, ON, USA). Membranes to be re-used were stripped by rinsing briefly with Milli-Q water, washing twice at 37 °C with 0.2 M sodium hydroxide/0.1% SDS for 15 min, and rinsing for 5 min at room temperature in 2× SSC. Before re-hybridization with a new probe, absence of prior signal was verified by adding detection reagent and re-exposure of membranes to film.

## 3. Results

### 3.1. Plasmid Transformation of R. buchneri and R. peacockii

*Rickettsia buchneri* and *R. peacockii* were successfully transfected with the shuttle vector pRAM18d carrying genes for rifampin or spectinomycin resistance and expression of a fluorescent protein [21].

**Figure 1:**
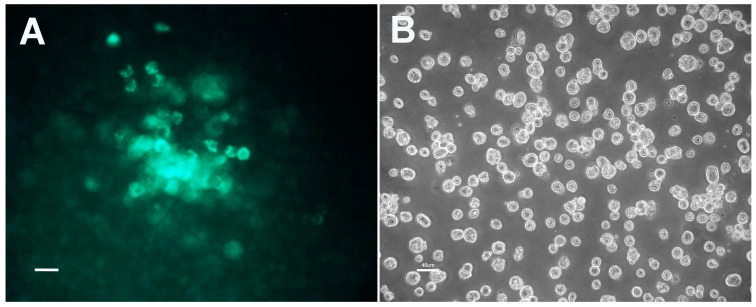

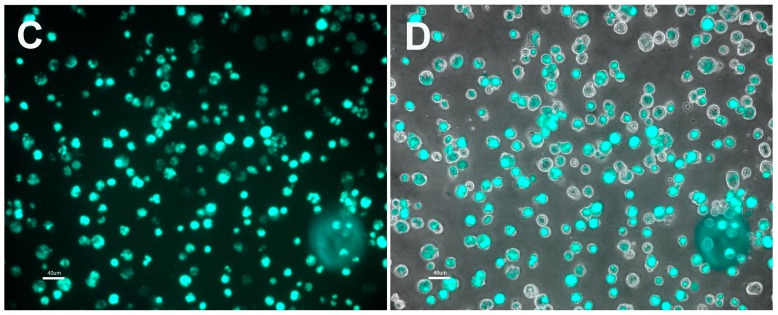
Images of *Rickettsia buchneri* ISO7 clone B8 transformed with shuttle vector pRAM18dRGA to express GFP_uv_. Transformants were isolated and grown in *Ixodes ricinus* embryonic cell line IRE11. Panel (**A**) Island of IRE11 cells containing transformed *R. buchneri* expressing GFP_uv_. Image collected 2.5 months after electroporation and rifampicin selection. Infected cells visualized using a fluorescein isothiocyanate (FITC) filter; Panel (**B**) Phase contrast microscopic appearance of IRE11 cells heavily infected with transformed *R. buchneri*. Transformed *R. buchneri* were in the 23rd serial transfer when imaged. Transformants were maintained in cell cultures at high density (1–5 × 10^6^ cells/mL) and were diluted for this image. Infected cells detach; (**C**) Same field as shown in panel (**B**) but visualized using fluorescence microscopy with FITC filter; (**D**) Composite image made by merging images shown in Panels (**B**,**C**). All images taken with a Nikon Diaphot fluorescence microscope. Bar equals 40 µm in all panels.

To transform *R. buchneri* to express a fluorescent protein, we used a 10,302-bp shuttle vector pRAM18dRGA [21] based in part on an 18,497-bp plasmid, pRAM18, present in *C.* R. amblyommii AaR/SC [31]. Clone ISO7-B8 was electroporated with pRAM18dRGA and seeded onto an IRE11 cell layer. Rifampin selection was maintained for six weeks, and discontinued when no fluorescent rickettsiae were detected. One month later, we noted small clusters of cells infected with rickettsiae expressing GFPuv (Figure 1, Panel A). Transformed *R. buchneri* replicated slowly within tick cells, and required two months to grow and spread within the cell layer before the first transfer to fresh cells was done. Transformed *R. buchneri* are presently in the 23rd subculture and can be transferred once a month by seeding infected cells (Figure 1, Panels B, C and D) onto a fresh IRE11 cell layer at a 1:5 dilution, indicating that *R. buchneri* has a doubling time of a week or more.

**Figure 2:**
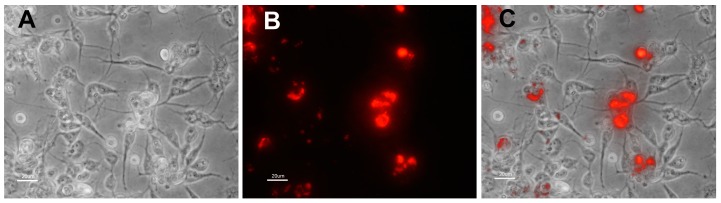
Image of *Ixodes scapularis* cells (ISE6) infected with *Rickettsia peacockii* expressing the far red fluorescent protein mKATE. Panel (**A**) Phase contrast microscopic appearance of ISE6 cells infected with transformed *R. peacockii*. Transformed *R. peacockii* were in the 23rd serial transfer when image was collected. Transformant is maintained in cell layers seeded at high cell density (1–5 × 10^6^ cells per mL); (**B**) Same field as shown in panel B but visualized using fluorescence microscopy with TRITC filter; (**C**) Composite image made by merging images shown in Panels (**A**,**B**). All images taken using a Nikon Diaphot fluorescence microscope. Bar equals 40 µm in all panels.

In contrast, *Rickettsia peacocki* was readily transformed using a shuttle vector carrying genes coding for spectinomycin resistance and the far-red fluorescent protein mKATE (pRAM18dSFA[MCS]) (Figure 2), or GFPuv (pRAM18dSGK[MCS]) [21]. Both red and green fluorescent *R. peacockii* transformants replicated faster than *R. buchneri*. Colonies of transformants were noted in cell layers at three weeks and transfers were made one month post electroporation. Transformants were maintained by subculturing transformed cells onto a fresh cell layer 1:50 (0.1 mL per 5 mL culture) every two to three weeks. We were unable to obtain *R. peacockii* transformants with the shuttle vector pRAM18dRGA and rifampin selection.

### 3.2. Characteristics of Transformed R. buchneri and R. peacockii in Tick Cell Culture

Transformants of both species displayed growth characteristics similar to those shown by wild type strains. Foci of cells infected with rickettsiae were readily evident in infected cell layers. In contrast, the formation of plaques, a feature of pathogenic rickettsiae, was not observed. Transformed symbionts grew in the cytoplasm, usually within clusters that expanded to eventually fill the cytoplasm and caused loss of host cell pseudopodia. Both species adhered poorly to host cells and cell free rickettsiae accumulated in the culture medium between transfers.

### 3.3. Identification of Shuttle Vector pRAM18dRGA and Native Plasmids pREIS1-3 in R. buchneri Transformant and Clone B8

**Figure 3:**
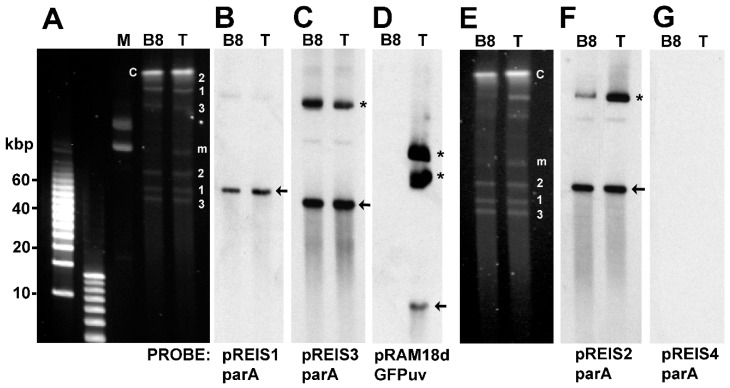
Identification of shuttle vector pRAM18dRGA and native plasmids pREIS1-3 in *R. buchneri* transformant and clone B8 by pulsed field gel electrophoresis (PFGE) and Southern blot (SB) analysis. Native *R. buchneri* (B8) and pRAM18dRGA (T) plasmids in REIS. A. PFGE gel; B & C. SB of gel in panel A probed with digoxygenin labeled *parA* probes specific for pREIS1 and pREIS3, respectively; D. SB of panel A probed with GFPuv; E. PFGE gel; F & G. SB of gel in panel A probed with digoxygenin labeled *parA* probes specific for pREIS2 and pREIS4, respectively. Asterisks mark putative linear monomer of each plasmid and arrows indicate their conformational isomers. IRE11 mitochondrial DNA—m. Chromosomal DNA—C. Linear DNA marker positions are to the left of panel A.

We used Pulsed field gel electrophoresis (PFGE) and Southern blots to detect the presence of the native pREIS plasmids (pREIS1, 2 and 3) in the B8 clone of *R. buchneri* and confirm the presence of shuttle vector plasmids in the transformants. Cell free *R. buchneri* were embedded in agar plugs and lysed with proteinase K, sodium lauryl sarcosine, and EDTA to release plasmid and chromosomal DNA. PFGE separated plasmids and shuttle vector from the larger *R. buchneri* chromosome. Ethidium bromide stained PFGE gels (Figure 3, Panels A and E) showed the presence of *I. scapularis* mitochondria (m), *R. buchneri* chromosome (C), and multiple plasmid bands in both the B8 clone (lane B8) and the GFP expressing transformant (lane T). The 10 kbp shuttle vector, pRAM18dRGA, was not apparent in the ethidium bromide stained PFGE gel. Southern blotting using digoxigenin-labeled probes revealed the location of the pREIS plasmids and the shuttle vector within the gels (Figure 3, Panels B, C, D, and F). Probes containing the *parA* gene of pREIS1 (55 kbp), 2 (67 kbp), and 3 (50 kbp) were used to detect the native plasmids. In both clone B8 and the transformant the three pREIS plasmids were present and clearly visible in stained blots (Figure 3, Panels B, C, and F). The pREIS 4 (34 kbp) is not present in clone B8 [3] and the southern blot using the pREIS *parA* probe confirmed the absence of pREIS4 in both the parent and the transformant (Figure 3, Panel G). Monomers (arrows) of each plasmid and their conformational isomers were present (asterisks). To check for the presence of the shuttle vector (pRAM18dRGA) in the transformant, the blot was stripped and re-probed with a probe containing the *GFPuv* gene. The smaller 10 kbp pRAM18dRGA and its conformational isomers was clearly present in the transformant (T) but not in clone B8 (Figure 3, lane D). In summary, these results indicate that the transformant retained the original 3 pREIS plasmids found in clone B8, pREIS1-3, and acquired the intact shuttle vector pRAM18dRGA.

### 3.4. Identification of pRPR and pRAM18dSGA in R. peacockii and Transformants

**Figure 4:**
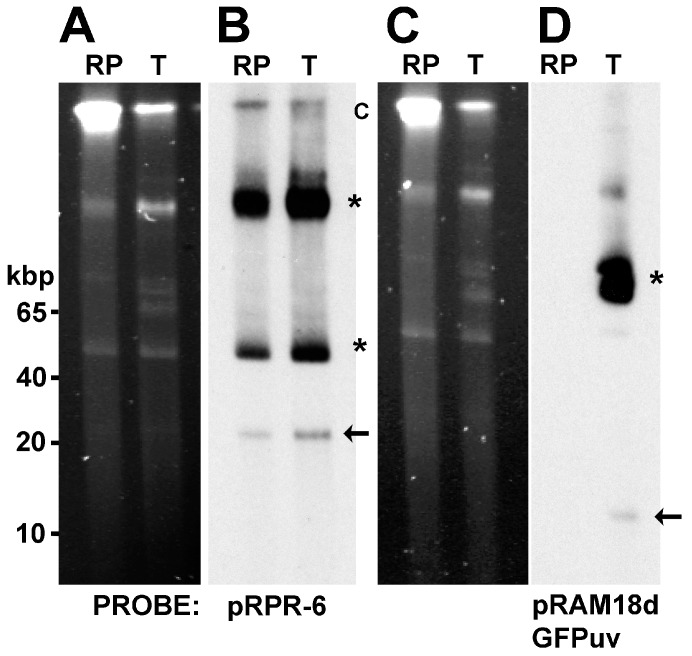
Identification of plasmids pRPR and pRAM18d in untransformed *R. peacockii* (RP) and transformant (T) by PFGE and Southern blot (SB) analysis. Panels A and C. PFGE gels showing prominent chromosomal band and several plasmid bands; Panels B and D. Southern Blots of gels shown in panels A and C; Panel B. Southern Blot probed with digoxygenin labeled pRPR-6 (probe specific RP_p06 gene in pRPR). Arrow points to linear monomer of native 26 kb plasmid of *R. peacockii*; Panel D. Southern blot probed with digoxygenin labeled GFPuv probe. Note probe binding in lane T only and absence of reactivity in RP lane. Arrows mark putative linear monomer of each plasmid and asterisks indicate their conformational isomers; Panel B. Note pRPR-6 probe reactivity in both RP and T. Linear DNA marker positions are to the left of panel A.

PFGE and Southern blots demonstrated the presence of the native pRPR plasmid [11] in *R. peacockii* and confirmed the presence of shuttle vector pRAM18dSGA plasmid in the transformant. Ethidium bromide stained PFGE gels (Figure 4, Panels A and C) showed the presence of the *R. peacockii* chromosome (C) and multiple smaller bands in both the native *R. peacockii* (lane RP) and the GFP expressing transformant (lane T). The 10 kbp shuttle vector, pRAM18dSGA, was not apparent in the ethidium bromide stained PFGE gel. As with *R. buchneri*, Southern blots using digoxigenin-labeled probes revealed the location of the pRPR plasmid and the shuttle vector within the gels (Figure 4, panels B and D). A pRPR-6 probe was used to detect the native plasmid pRPR. In both untransformed and transformed *R. peacockii* the pRPR plasmid was present and clearly visible in stained blots (Figure 4, Panels B and D). Monomers (arrows) of each plasmid and the more predominant conformational isomers were present (asterisks) in both pRPR and pRAM18dSGA. Again, these results indicate that the transformant retained its original plasmid, pRPR, and acquired the intact shuttle vector pRAM18dSGA.

### 3.5. Transposon Mutagenesis (HIMAR1 A7) of R. peacockii

**Figure 5:**
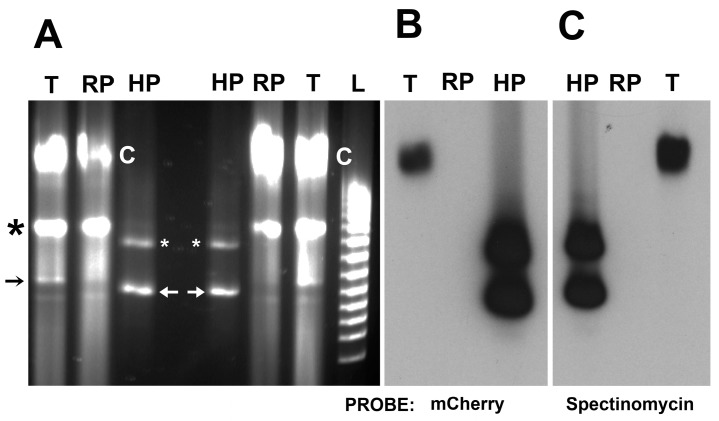
PFGE and Southern blot analysis of *R. peacockii* mutant obtained by transposon mutagenesis (HIMAR A7). Panel A. PFGE comparison of transformant (T) with wild type *R. peacockii* (RP). Black arrows point to position of linear monomers of the native 26 kbp pRPR and black asterisk indicates position of its conformational isomers. Lane L contains 5 kb ladder. HP lanes contain the 8.4 kb *Himar1* plasmid (pCis mCherry-SS HIMAR1 A7) encoding mCherry and spectinomycin resistance (*aadA*) genes. White arrows point to position of supercoiled of pCis mCherry-SS HIMAR1 A7 and asterisks to its multimers; Panel B. Southern blot probed with digoxygenin labeled mCherry probe. Note probe binding in lane T to the region of the chromosome, to supercoiled and multimeric forms of pCis mCherry-SS HIMAR1 A7 plasmid bands in the HP lane, and absence of reactivity with the 26 kb pRPR; Panel C. Southern blot probed with the digoxygenin labeled spectinomycin resistance gene. The probe bound in lane T to the region of the chromosome, labeled the *himar1* plasmid in the HP lane, but did not react with the 26 kb pRPR in lane RP. This is consistent with results shown in Panel B.

We transformed *R. peacockii* to express either mCherry or GFP_uv_ using the plasmid pCis mCherry-SS HIMAR1 A7 or pCis uv-SS HIMAR1 A7 [20,22]. As decribed above, both included the *A. marginale* promoter tr driving expression of the fluorescent proteins GFPuv or mCherry and spectinomycin resistance flanked by the left and right Himar1 transposon repeats. We grew the rickettsiae in ISE6 cells under selection with the clinically irrelevant antibiotic spectinomycin and successfully isolated *R. peacockii* mutants that express GFP or mCherry (Figure 5). PFGE followed by Southern blotting using mCherry and spectinomycin digoxygenin labeled probes revealed that the transposon were inserted into the chromosome. Further Southern blot analysis using infrequent cutting endonucleases (BglII, EcoRI, HindIII, HpaI, EcoRV, EcoRI, and HindIII) demonstrated a single insertion into the *R. peacockii* chromosome (data not shown). The genomic insertion site was determined by plasmid rescue cloning [30]. Transposon insertion sites in the *R. peacockii* (*R. peacockii* str. Rustic complete genome) Himar1 transformant mapped to CP001227.1 bp 544,605/544,606. This is an intergenic insertion downstream of a hypothetical protein gene (a predicted transcriptional regulator, bp 544,040–544,534) and upstream of an aspartate kinase gene (bp 544,818–546,023) [11].

**Figure 6:**
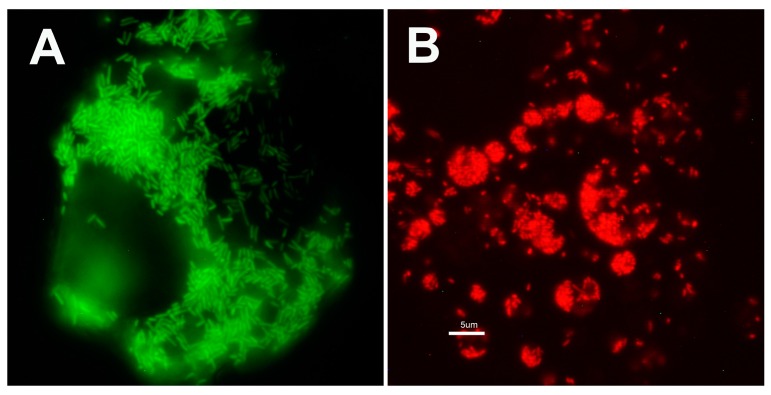
Images of *Ixodes* cells infected with *Rickettsia buchneri* and *Rickettsia peacockii* expressing GFP_uv_ and mCherry. Panel (**A**) Fluorescence microscopic appearance of IRE11 cells infected with GFP_uv_ expressing *R. buchneri*. Transformed *R. buchneri* were in the XX serial transfer when image was collected. Transformant is maintained in cell layers seeded at high cell density (1–5 × 10^6^ cells per mL). Rickettsiae were visualized using fluorescence microscopy with FITC filter; (**B**) Fluorescence microscopic appearance of ISE6 cells infected with mCherry expressing *R. peacockii*. Rickettsiae were visualized using fluorescence microscopy with TRITC filter. All images taken using an upright Nikon Eclipse E400Diaphot fluorescence microscope. Bar equals 5 µm. Both images were collected at the same magnification.

In contrast to the *R. peacockii* transformed using shuttle vectors, the expression of mCherry and GFP_uv_ was significantly lower in transposon mutants. Thus, mutants were visualized by fluorescence microscopy using a Nikon Eclipse E400 upright or an Olympus spinning disk DSU/BX60 confocal microscope (Figure 6).

## 4. Discussion

In this communication, we report the successful genetic transformation of two rickettsiae widely recognized as non-pathogenic mutualistic symbionts, *R. buchneri* and *R. peacockii*. The beneficial effects of these rickettsiae on their host ticks range from protection of the tick from super infection by pathogenic rickettsiae [1] to nutritional supplementation [2]. We have demonstrated that fluorescent protein and antibiotic resistance genes can be delivered to *R. buchneri* and *R. peacockii* on shuttle vectors developed from *R. amblyommi*, a species that carries multiple native plasmids. We have also demonstrated that rickettsial symbionts can also acquire and maintain these low-copy-number plasmids, each rather unique in their genetic makeup, apparently without the loss of their native plasmids [2,21,30,32]. In addition, we have shown that *himar1*, a broad range *Mariner* element, was useful for the insertion of fluorescent protein and antibiotic resistance genes into the genome of *R. peacockii*. Rickettsial symbionts of ticks require tick cells for replication and selection of transformants that are rare and slow growing. They benefited greatly from the use of tick host cells that themselves grow slowly and can be maintained at high density for extended periods. While *R. peacockii* has a wider host cell range in vitro [33], *R. buchneri* is more fastidious and we have found only two *Ixodes* cell lines, ISE6 and IRE11, from *Ixodes* ticks that are suitable for isolating and growing *R. buchneri*.

We have identified factors that hindered our progress and limited the number of transformants that we were able to isolate. Most noticeable was the low yield of transformants from a single electroporation. The low yield exacerbated the length of time required for transformants to replicate to detectable levels. Several weeks were required for the detection of fluorescent bacterial colonies by microscopy and there were few colonies within the cell layer. This indicated that very few of the symbionts had survived electroporation and/or acquired the transforming plasmid. This was especially apparent when we electroporated *R. buchneri*, a rickettsia having a long doubling time, with a shuttle vector. The isolation of *himar1* transformed *R. peacockii* was met with limited success. The finding that the *himar1 R. peacockii* transformant population contained a single insertion site indicated that the population was clonal and arose from a single survivor of the electroporation and selection conditions that were used. Targeting specific genes via site directed mutagenesis and achieving “saturated mutagenesis” depends on our ability to define and improve important transformation parameters. Improved transformation conditions are necessary before we can apply genome-wide mutational analysis of rickettsial symbionts. The challenge that remains is to develop a homologous recombination system, but progress being made with other rickettsiae [34,35,36] may assist us.

Our results demonstrate that studies on the experimental parameters influencing the isolation of *R. buchneri* and *R. peacockii* mutants are needed, as the reasons for the low yield remain to be identified. Low penetration of plasmid DNA into the symbionts, massive killing due to electroporation conditions and excessive antibiotic selection are potential factors that need to be examined. Recently, Driskell et al. [37] used fluorescence activated cell sorting to harvest cells infected with *R. prowazekii* expressing fluorescent reporter proteins. To reduce time and variability, frozen stocks of electrocompetent rickettsiae were used. They detected transformants as early as one week after electroporation. Shuttle vector (transforming DNA) concentration was identified as being critical to detecting transformants as soon as a few days after transfecting cells with *R. prowazekii.* Also needed is research on the effects of selection pressure (concentration and timing of antibiotic selection) and electroporation conditions. The use of non-pathogenic rickettsial symbionts, as opposed to *R. prowazekii*, a BSL3 pathogen, will further facilitate progress in this area.

These mutant endosymbionts expressing fluorescent proteins will be useful for future studies on rickettsia-host interactions in vivo and in vitro. Shuttle vectors provide a way to introduce large and diverse constructs into rickettsial symbionts, circumventing the size limitations imposed by the *himar1* transposon [29]. Furthermore, the shuttle vector system will also be useful for complementation assays aimed at examining the function of the wide array of genes that are defective in these two species. They will also enable paratransgenic studies using transformed rickettsial symbionts expressing anti-pathogen factors. Recent studies show that book lice (*Liposcelis bostrychophila*) and the tick *Ixodes pacificus* can be cured of endosymbiotic rickettsiae using heat or antibiotics [13,38,39], potentially setting the stage for future research aiming to reintroduce *R. buchneri* and *R. peacockii* transformants back into aposymbiotic host ticks.

## 5. Conclusions

We have made progress in the genetic manipulation of two rickettsia species being used as models for mutualistic symbionts, *R. buchnerii* and *R. peacockii*.

Transformed endosymbiotic rickettsiae maintained multiple plasmids, including shuttle plasmids carrying fluorescent protein and antibiotic resistance transgenes.

Future studies are needed to define the experimental parameters that influence the isolation of *R. buchneri* and *R. peacockii* mutants.

## Figures and Tables

**Table 1:** Plasmid constructs used for the transformation of *Rickettsia buchneri* and *Rickettsia peacockii*.

Construct	MW (bp)	Fluoro-Chrome	Antibiotic Selection	Symbiont
**Shuttle Vector Plasmids**				
pRAM18dRGA	10,248	GFP_uv_	Rifampin	*R. buchneri*
pRAM18dRGA[MCS]	10,309	GFP_uv_	Rifampin	*R. buchneri*
pRAM18dSGA[MCS]	10,736	GFP_uv_	Spectinomycin	*R. peacockii*
pRAM18dSGK(23)[MCS]	11,525	GFP_uv_	Spectinomycin	*R. peacockii*
pRAM18dSFA[MCS]	10,829	mKate	Spectinomycin	*R. peacockii*
**Himar I Transposase-Transposon Plasmid**				
pCis mCherry-SS HIMAR1 A7	8423	mCherry Spec.	Spectinomycin	*R. peacockii*
